# Less Waste on Waist Measurements: Determination of Optimal Waist Circumference Measurement Site to Predict Visceral Adipose Tissue in Postmenopausal Women with Obesity

**DOI:** 10.3390/nu10020239

**Published:** 2018-02-20

**Authors:** Radhika V. Seimon, Anthony L. Wild-Taylor, Alice A. Gibson, Claudia Harper, Sally McClintock, Hamish A. Fernando, Michelle S. H. Hsu, Felipe Q. da Luz, Shelley E. Keating, Nathan A. Johnson, Stuart M. Grieve, Tania P. Markovic, Ian D. Caterson, Nuala M. Byrne, Amanda Sainsbury

**Affiliations:** 1 The Boden Institute of Obesity, Nutrition, Exercise & Eating Disorders, Sydney Medical School, Charles Perkins Centre, The University of Sydney, Camperdown, NSW 2050, Australia; anthony.wildtaylor@sydney.edu.au (A.L.W.-T.); alice.gibson@sydney.edu.au (A.A.G.); claudia.harper@sydney.edu.au (C.H.); sally.mcclintock@sydney.edu.au (S.M.); hamish.fernando@sydney.edu.au (H.A.F.); michelle.hsu@sydney.edu.au (M.S.H.H.); felipe.quintodaluz@sydney.edu.au (F.Q.d.L.); nathan.johnson@sydney.edu.au (N.A.J.); tania.markovic@sydney.edu.au (T.P.M.); ian.caterson@sydney.edu.au (I.D.C.); amanda.salis@sydney.edu.au (A.S.); 2 School of Psychology, Faculty of Science, The University of Sydney, Camperdown, NSW 2050, Australia; 3 School of Human Movement and Nutrition Sciences, Centre for Research on Exercise, Physical Activity and Health, The University of Queensland, Brisbane, QLD 4072, Australia; s.keating@uq.edu.au; 4 Faculty of Health Sciences, The University of Sydney, Sydney, NSW 2050, Australia; 5 Sydney Translational Imaging Laboratory, Heart Research Institute, Charles Perkins Centre, The University of Sydney, Camperdown, NSW 2050, Australia; stuart.grieve@sydney.edu.au; 6 Department of Radiology, Royal Prince Alfred Hospital, Camperdown, NSW 2050, Australia; 7 Metabolism & Obesity Services, Royal Prince Alfred Hospital, Camperdown, NSW 2050, Australia; 8 School of Health Sciences, College of Health and Medicine, University of Tasmania, Launceston, TAS 7250, Australia; nuala.byrne@utas.edu.au

**Keywords:** waist circumference, magnetic resonance imaging, visceral adipose tissue, obesity

## Abstract

With obesity being a leading cause of preventable death, it is vital to understand how best to identify individuals with greater risk of metabolic disease, especially those with high visceral adipose tissue (VAT). This study aimed to determine whether three commonly used waist circumference (WC) measurement sites could provide accurate estimations of VAT, as determined by magnetic resonance imaging (MRI), which is a gold standard for measuring VAT, in postmenopausal women with obesity. VAT volume was measured by MRI of the total abdomen in 97 women aged 57.7 ± 0.4 years (mean ± SEM), mean body mass index 34.5 ± 0.2 kg/m^2^. WC was measured at the midpoint between the lowest rib and the iliac crest (WC_mid_), the narrowest point of the torso (WC_narrow_), and at the level of the umbilicus (WC_umbilicus_). WC differed significantly according to measurement site, with WC_narrow_ (102.1 ± 0.7 cm) < WC_mid_ (108.3 ± 0.7 cm) < WC_umbilicus_ (115.7 ± 0.8 cm) (*p* < 0.001). WC_mid_, WC_narrow_ and WC_umbilicus_ were all significantly correlated with VAT, as measured by MRI (*r* = 0.581, 0.563 and 0.390, respectively; *p* < 0.001 for all), but the relationships between WC_mid_ or WC_narrow_ and VAT determined by MRI were stronger than for WC_umbilicus_. Measurement of either WC_mid_ or WC_narrow_ provides valid estimates of VAT in postmenopausal women with obesity, with WC_narrow_ being favoured in light of its greater ease and speed of measurement in this population.

## 1. Introduction

Obesity, which can be defined as an excess of body fat, is an ever-growing public health crisis related to the development of many disturbances, including type 2 diabetes and cardiovascular disease [1,2]. Body fat is distributed in two main compartments with different metabolic characteristics: subcutaneous adipose tissue (SAT) and visceral adipose tissue (VAT). However, the metabolic risks associated with obesity, such as impaired glucose and lipid metabolism, diabetes, insulin resistance [3,4,5], hypertension [6] and metabolic syndrome [7], have been attributed to increases in VAT [8].

An accurate measure of VAT requires the use of gold-standard imaging techniques, such as computed tomography (CT) or magnetic resonance imaging (MRI) [9]. However, while MRI, avoids the radiation exposure encountered with CT, both CT and MRI are relatively inaccessible, expensive, and image analysis is labour intensive [10]. Dual-energy X-ray absorptiometry (DXA) is an alternative method for estimating VAT [11] that has been shown to correlate strongly with VAT measured using CT [12,13,14,15,16] and MRI [17,18]. DXA delivers a minimal radiation dose and is less costly and time-consuming than CT or MRI for measuring VAT [19]. However, DXA too requires expensive equipment and trained technicians [19]. 

A simple index of VAT is waist circumference (WC), which provides a recognised estimate of abdominal adiposity and metabolic risk [20]. There are different WC measurement sites that are routinely used [21]. For example, a literature review identified 14 different descriptions of the site for WC measurements [22]. However, there is no consensus regarding the optimal WC measurement site [23]. The World Health Organisation recommends measuring WC at the midpoint between the bony landmarks of the lowest rib and the top of the iliac crest (WC_mid_) [24]. The Anthropometric Standardization Reference Manual (developed in 1985 by experts attending the Anthropometric Standardization Conference) recommends measuring WC at the narrowest point of the torso (WC_narrow_) [25]. In contrast, some research studies have measured WC at the level of the umbilicus (WC_umbilicus_) [26,27], or have failed to describe the WC measurement site used at all [28,29]. A systematic review looking at 120 studies of WC measurement sites and morbidity or mortality found that most WC measurements were performed at WC_mid_ (30%), WC_narrow_ (27%) or WC_umbilicus_ (29%) [26]. Further, in the few studies that have investigated the relationship between VAT (using the gold standard techniques of CT and MRI) and WC, they did so only in zero [30], one [31,32,33] or two [34] of the three common WC measurement sites.

To identify those with greater levels of VAT and therefore, greater risk of metabolic disease, especially in higher risk populations, such as older adults or those with obesity, we aimed to determine which of the three common measures of WC (WC_mid_, WC_narrow_ and WC_umbilicus_) is the better predictor of VAT, as determined by the gold standard method of MRI, specifically in postmenopausal women with obesity.

## 2. Materials and Methods

### 2.1. Ethics Statement and Participants

The study was approved by the Sydney Local Health District Ethics Committee (Royal Prince Alfred Hospital Zone) and registered with the Australia and New Zealand Clinical Trials registry (number 12612000651886). All participants provided informed, written consent prior to participation. 

Ninety-seven women, aged 57.7 ± 0.4 years (mean ± SEM), with a mean body mass index (BMI) of 34.4 ± 0.3 kg/m^2^ participated in this study. Participants were at least five years postmenopausal at the time of recruitment, and were predominately Caucasian (*n* = 92). Participants were recruited as part of a broader study: the randomised controlled TEMPO Diet Trial (Type of Energy Manipulation for Promoting optimum metabolic health and body composition in Obesity). All participants were required to be weight stable (±2 kg) for ≥6 months, and sedentary (defined as <3 h of structured physical activity per week). Exclusion criteria were not being ambulatory, or having osteoporosis, hyperthyroidism or hypothyroidism, diabetes mellitus, any loose metal in the body (e.g., pacemaker or bullet) that is contraindicated for MRI for safety reasons, or which may result in artefacts in medical imaging, tobacco use, alcohol or drug dependency.

### 2.2. Measurements

#### 2.2.1. Anthropometry

Body weight (Tanita BWB-800 digital scale, Wedderburn Pty. Ltd., Sydney, Australia) and standing height (Harpenden Stadiometer, Holtain Ltd., Crymych, UK) were both measured twice to the nearest 0.1 kg and 0.1 cm, respectively. If the difference between the measurements was >0.5 kg for body weight or >0.5 cm for standing height, a third measurement was taken. The average of the two measurements (or the average of the two closest measurements if a third measurement was taken) was recorded as the result. Participants were dressed in leggings and a sports bra and were not wearing shoes during measurement.

#### 2.2.2. MRI

Abdominal fat volumes (i.e., VAT) were measured by MRI, with participants lying in a supine position in a hospital gown, having removed any metal accessories. Axial T1-weighted fast field echo images were acquired with a 3.0T MRI scanner (the Discovery MR750 3.0T model from GE Healthcare, Milwaukee, WI, USA), from diaphragm to pelvis (repetition time = 3.8 ms, echo time = 2.1 ms, flip angle = 12°), with a slice thickness of 10 mm and an inter-slice gap (i.e., the distance between the surfaces of adjacent slices) of 10 mm. Images were acquired during suspended end-expiration, with a breath-hold duration of approximately 15–18 s per acquisition. Following the scan, all image slices from the base of the lungs to the pelvic floor were segmented manually. VAT was quantified using the Region Growing mode of the analysis software (SliceOMatic Version 5.0 rev-6b, Tomovision Inc., Montreal, QC, Canada), with thresholds adjusted manually, as required. The software automatically calculated the surface area of each slice by multiplying the number of pixels tagged by the surface area of one pixel. The inter-slice volume (i.e., the volume of the inter-slice gap) was extrapolated using a cone formula that considered the surface area of the superior and inferior surfaces of the inter-slice gap, as well as the thickness of the inter-slice gap. The total volume of each of the inter-slice gaps was then added to the total volume of each of the slices (surface area × slice thickness) to calculate the total abdominal volumes of VAT. All analyses were conducted by the same researcher (ALWT).

#### 2.2.3. WC

WC was measured to the nearest 0.1 cm, directly on skin, using a narrow, flexible and inelastic steel tape (Lufkin W606PM, Apex Tool Group, North Carolina, USA). Participants were dressed in leggings and a sports bra. WC was measured at the three most commonly used sites worldwide [26]; WC_mid_, WC_narrow_ and WC_umbilicus_. Participants were asked to breathe normally and to stand with their weight evenly distributed and their arms crossed over their shoulders during measurements. At each measurement site, two measurements were taken, and if the difference between the measurements was >1 cm, a third measurement was taken. The average of each of the two measurements at each site (or the average of the two closest measurements if a third measurement was taken) was recorded as the result. Measurements were taken by the same two researchers (SM and HAF) throughout the study. The inter-observer coefficient of variation (CV) in a subset of five participants was 1.44% for WC_mid_, 0.66% for WC_narrow_, and 0.69% for WC_umbilicus_. The intra-observer CV for the two measurements on all 97 participants was 0.27% for WC_mid_, 0.32% for WC_narrow_ and 0.23% for WC_umbilicus_. 

### 2.3. Statistical Analysis

All data are presented as means ± standard error of the mean (SEM), unless otherwise stated. A Shapiro–Wilk test of normality demonstrated the normal distribution of all data used in this study. Comparisons between the three different WC measurement sites were performed using one-way repeated measures ANOVA plus a post hoc test for multiple comparisons. Pearson correlations were used to assess the relationships between VAT volume and different WC measurement sites, or BMI. A Fisher’s z-transformation was used to determine if the correlation coefficients between each of the relationships were significantly different from each other. Statistical significance was accepted as *p* < 0.05. Statistical analyses were performed using SPSS Version 24 (IBM SPSS Statistics, IBM Corporation, New York, NY, USA).

## 3. Results

Descriptive statistics for the participants in this study are presented in Table 1.

**Figure 1:**
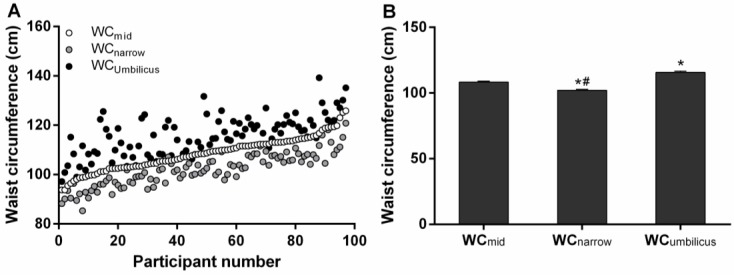
Waist circumference (WC) measurement at different sites. WC at the midpoint (WC_mid_), narrowest point (WC_narrow_) and umbilicus (WC_umbilicus_) for each individual participant (**A**); and as means ± SEM (**B**), in postmenopausal women with obesity (*n* = 97). * *p* < 0.05 versus WC_mid_, # *p* < 0.05 versus WC_umbilicus._

The three different WC measurement sites produced significantly different results, with WC_narrow_ < WC_mid_ < WC_umbilicus_ (Table 1, Figure 1). The greatest differences were observed between WC_narrow_ and WC_umbilicus_ (13.5 ± 0.6 cm, *p* < 0.05), then between WC_mid_ and WC_umbilicus_ (7.1 ± 0.6 cm, *p* < 0.05), with the smallest difference being between WC_narrow_ and WC_mid_ (6.3 ± 0.3 cm, *p* < 0.05).

**Figure 2:**
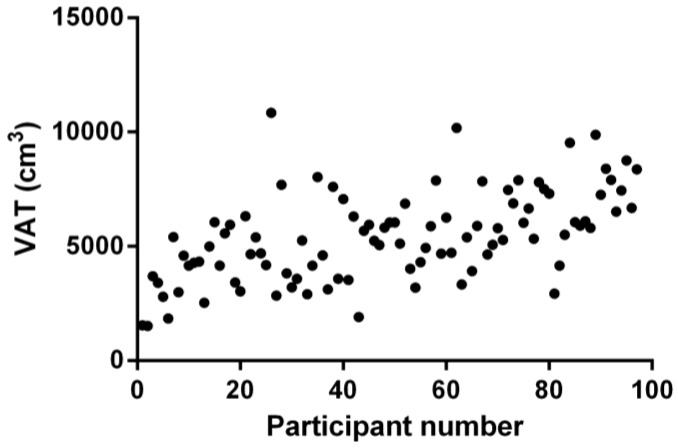
Visceral adipose tissue volume (VAT), as determined by magnetic resonance imaging for each of the postmenopausal women with obesity in this study (*n* = 97).

Despite the narrow BMI range of participants in this study (Table 1), in keeping with the selection criteria, there was significant variation in WC (Table 1, Figure 1) and VAT volumes (Table 1, Figure 2) across the cohort. These wide ranges of WC and VAT values are helpful for the current study, in order to demonstrate the relative utility of different WC measurement indexes.

**Figure 3:**
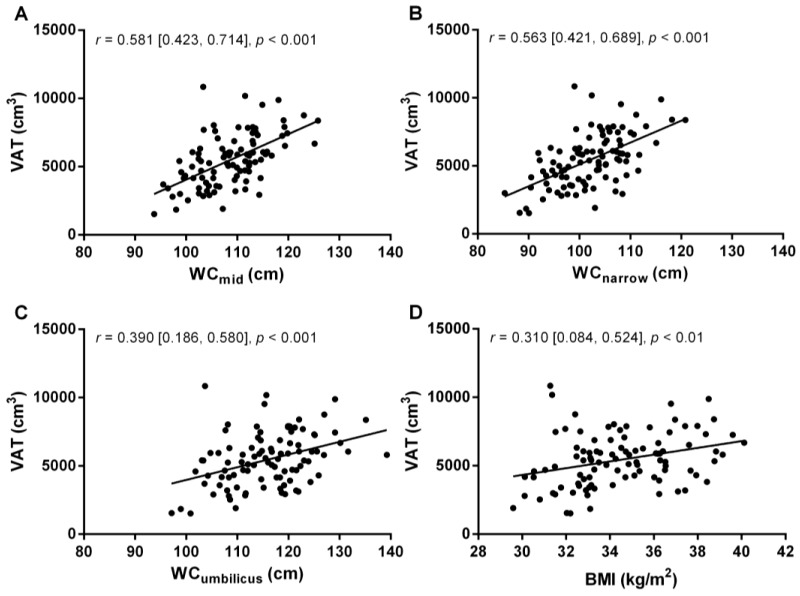
Visceral adipose tissue volume (VAT), measured by magnetic resonance imaging, versus waist circumference measured at the midpoint (WC_mid_, **A**); narrowest point (WC_narrow_, **B**) and umbilicus (WC_umbilicus_, **C**); as well as body mass index (BMI, **D**), in postmenopausal women with obesity (*n* = 97). Data on each panel represent correlation coefficients (*r*) as well as their 95% confidence intervals [lower limit, upper limit], and corresponding *p* values.

The three different waist circumference measures, and BMI, were significantly correlated with VAT, as determined by MRI (Figure 3). WC_mid_ and WC_narrow_ were both moderate correlates of VAT, while WC_umbilicus_ was a weak, and BMI a very weak, correlate of VAT (as shown by the correlation coefficients (*r*) and 95% confidence intervals in Figure 3).

There was no significant difference between the correlations of VAT–WC_mid_ and VAT–WC_narrow_ (*z* = 0.18, *p* = 0.86). The difference in correlations between VAT–WC_mid_ and VAT–WC_umbilicus_ approached, but did not reach statistical significance (*z* = 1.73, *p* = 0.08), as did the correlations between VAT–WC_narrow_ and VAT–WC_umbilicus_ (*z* = 1.55, *p* = 0.12). In contrast, there was a significant difference between the correlations of VAT–BMI and VAT–WC_mid_ (*z* = 2.35, *p* = 0.02) and VAT–BMI and VAT–WC_narrow_ (*z* = 2.17, *p* = 0.03), but there was no significant difference in correlations between VAT–BMI and VAT–WC_umbilicus_ (*z* = 0.63, *p* = 0.53). In addition, when comparing each of the three different WC measurement sites against one another, there was a strong correlation between WC_mid_ and WC_narrow_ (*r* = 0.91), which was significantly stronger than either of the correlations between WC_mid_ and WC_umbilicus_ (*r* = 0.715), or WC_narrow_ and WC_umbilicus_ (*r* = 0.702). This data, combined with the correlations between VAT and different WC measurement sites, shows that the most useful anthropometric indices of VAT volume are WC_mid_ or WC_narrow_.

## 4. Discussion

Our study investigated the correlation between VAT, as quantified by the gold standard method of MRI, and all three of the most common WC measurement sites [26], namely WC_mid_, WC_narrow_ and WC_umbilicus_. Our results indicate that WC_mid_ and WC_narrow_ are the most appropriate WC measurement sites for the estimation of VAT by MRI in postmenopausal women with obesity. Additionally, there was no significant difference in the correlations between WC_mid_ and WC_narrow_ with respect to their correlations with VAT, indicating that both are very similar measurement sites, while WC_narrow_ is easier and faster to measure than WC_mid_, as explained below.

While previous studies have investigated the relationship between VAT (determined by MRI or CT) and WC, they only investigated zero [30], one [31,32,33] or two [34] of the three common WC measurement sites. In the studies measuring only one WC site, they found WC_mid_ [31], WC_narrow_ [32,33], or WC measured at the halfway point between the L4 and L5 vertebrae [30], were all associated with VAT. The study that looked at two of the three common WC measurement sites (WC_narrow_ and WC_umbilicus_)—in an overweight to mildly obese population (average BMI 30.2 kg/m^2^, range 25–35 kg/m^2^)—found that VAT area measured by CT correlated slightly more strongly with WC_narrow_ (*r* = 0.63) than with WC_umbilicus_ (*r* = 0.57) [34]. The current study extends knowledge from this existent literature by directly comparing all three of the common WC measurement sites in the same study, thereby allowing for selection of the most appropriate WC measurement site(s) in postmenopausal women with obesity (WC_mid_ or WC_narrow_).

When considering the practicality of measuring WC_mid_ or WC_narrow_, WC_narrow_ is advantageous. This is because when measuring WC_mid_, the observer needs to identify and mark two anatomical landmarks (the bottom of the lowest rib and the top of the iliac crest), and then mark the midpoint between these two landmarks before WC can be measured. In contrast, when measuring WC_narrow,_ the observer can estimate the narrowest point of the torso by simply viewing the torso from the back prior to measuring WC. As such, measuring WC_narrow_ is less time consuming than WC_mid_. While some studies emphasise the need for bony landmarks to guide WC measurement [23], this can be problematic. The identification of both the rib and the iliac crest for the measurement of WC_mid_ can be difficult in people with increased adiposity. To identify these two landmarks, the observer may need to palpate the abdomen thoroughly, which can be intrusive and embarrassing for the subject [35]. In addition, at higher levels of adiposity, these landmarks might not be able to be located precisely, which would make the measurement imprecise, because inaccurate localization of the border of either the rib or the iliac crest can have a significant effect on the WC measured, thus leaving more room for error [22]. In summary, WC_narrow_ is not only a good estimate of VAT in postmenopausal women with obesity, but is also quicker and easier to measure than WC_mid_ and offers the advantage of avoiding the less practical procedure and potential imprecision of landmark identification. However, it should be noted that these results are valid for an environment where highly trained researchers take the measures, which may not apply to clinical practice.

This study showed that although WC_umbilicus_ was significantly correlated with VAT, as had also been shown in a previous study [34], it further showed that WC_umbilicus_ was of limited value in estimating VAT in postmenopausal women with obesity, when compared to WC_mid_ or WC_narrow_. This may be related to the relatively low position of this the umbilicus site in the abdomen. A study in overweight or obese participants found that the correlation between VAT area, measured by single-slice MRI, and VAT volume, measured by multi-slice MRI, was strongest higher in the abdomen compared to lower [36]. These findings suggest that WC measured in the upper abdomen may correlate more closely with VAT than WC measured in the lower abdomen. This can potentially explain the differences in the correlations between VAT and different measurement sites, and supports our findings where WC_mid_ and WC_narrow_—which are higher in the abdomen than WC_umbilicus_—are more strongly correlated with VAT than WC_umbilicus_. In addition, although measuring WC_umbilicus_ is the least time-consuming of all three methods, a limitation in the use of WC_umbilicus_ is the variability in its position, as the umbilicus is a soft tissue landmark [37]. A study looking at umbilical position and BMI in males and females found that the median position of the umbilicus was 0.88 cm, 1.20 cm, and 3.50 cm below the middle of the torso in people who had a BMI in the normal, overweight, or obese range, respectively [38]. While this change in the position of the umbilicus could indicate important VAT changes, the panniculus (the so called ‘apron of fat’ upon which the umbilicus sits) consists of subcutaneous fat, and therefore a drop in umbilical position is more likely to indicate subcutaneous fat accumulation than any change in VAT [39]. Therefore, one might expect WC_umbilicus_ to be a poorer correlate of VAT, especially in people with obesity, due to its varying position on the torso.

In this study we also looked at the correlation between VAT (as determined by MRI) and BMI in our 97 participants, and found that the correlation was very weak—significantly weaker than the relationship between WC_mid_ or WC_narrow_ and VAT. This finding underscores the strength and specificity of the correlation between WC_mid_ or WC_narrow_ and VAT. This finding is in contrast to large-scale population-based studies, including ~3,000–4,500 people, where BMI was significantly correlated with VAT, as measured by CT [4,40]. This highlights that although BMI may be an appropriate estimator of metabolic risk in large-scale population studies, it is not appropriate for evaluation of metabolic risk in smaller studies or in individuals [41].

Another finding from this study is that the site of WC measurement significantly influences the magnitude of the measurement, with WC_narrow_ < WC_mid_ < WC_umbilicus_ in our population of postmenopausal women with obesity. This finding is consistent with previous investigations suggesting that WC measurements made using different protocols are not all comparable [22,34,42]. For instance, in a study of WC measurements taken at four sites in 57 women, there were significant differences between all measurement sites (WC_narrow_ < WC immediately below the lowest rib < WC_mid_ < WC immediately above the iliac crest; *p* < 0.05 for all comparisons) [22]. The current study advances on existing findings by showing—within the same study—that even the three commonly used WC measurement sites are significantly different from each other, even in a relatively homogenous population of postmenopausal women with obesity. This calls into question the practice of not reporting the specific site at which WC was measured, as in some publications [28,29].

This study has several limitations and strengths. Selection criteria for the present study were narrow, and while this limits the ability to generalise the results to other population groups, it also strengthens the findings by making them specific to a high-risk population of postmenopausal women with obesity—a population where WC measurement site differences were relatively unknown. Another limitation of our study is that the WC measures were not investigated under conditions of weight change. In addition, a potential source of measurement error for all WC sites is incorrectly positioning the tape measure on the subject’s body, however, this was minimised in the current study with the use of only two researchers assessing WC. In addition, the heavy-duty tape measure used in our study was flexible, inelastic, and firm, making it easy to place around the trunk region of the body in the same plane. Another strength of our study was that data variability was reduced by the consistent analysis of MRI scans by one researcher for the whole study.

In conclusion, measurement of WC_mid_ or WC_narrow_ are accurate, practical and cost-effective means of estimating VAT in postmenopausal women with obesity, with WC_narrow_ offering several advantages for ease, speed and precision of measurement over WC_mid_.

## Figures and Tables

**Table 1:** Clinical and anthropometric characteristics of participants.

Age (years)	57.7 ± 0.4 (45–65)
Weight (kg)	91.1 ± 0.9 (76.6–116.4)
BMI (kg/m^2^)	34.5 ± 0.2 (29.6–40.1)
Waist circumferences (cm)	
WC_mid_	108.3 ± 0.7 (93.8–125.8)
WC_narrow_	102.1 ± 0.7 (85.3–120.8)
WC_umbilicus_	115.7 ± 0.8 (97.2–139.3)
VAT (cm^3^)	5,430 ± 200 (1510–10,840)

Data are presented as means ± SEM (range) of 97 postmenopausal women. BMI, body mass index; WC_mid_, waist circumference measured at the midpoint; WC_narrow_, WC measured at the narrowest point; WC_umbilicus_, WC measured at the umbilicus; VAT, visceral adipose tissue volume (measured by magnetic resonance imaging).

## References

[1] Kelly T., Yang W., Chen C.S., Reynolds K., He J. (2008). Global burden of obesity in 2005 and projections to 2030. Int. J. Obes..

[2] Afshin A., Forouzanfar M.H., Reitsma M.B., Sur P., Estep K., Lee A., Marczak L., Mokdad A.H., Moradi-Lakeh M., Naghavi M. (2017). Health Effects of Overweight and Obesity in 195 Countries over 25 Years. N. Engl. J. Med..

[3] Ritchie S.A., Connell J.M. (2007). The link between abdominal obesity, metabolic syndrome and cardiovascular disease. Nutr. Metab. Cardiovasc. Dis..

[4] Fox C.S., Massaro J.M., Hoffmann U., Pou K.M., Maurovich-Horvat P., Liu C.Y., Vasan R.S., Murabito J.M., Meigs J.B., Cupples L.A. (2007). Abdominal visceral and subcutaneous adipose tissue compartments: Association with metabolic risk factors in the Framingham Heart Study. Circulation.

[5] Goodpaster B.H., Krishnaswami S., Resnick H., Kelley D.E., Haggerty C., Harris T.B., Schwartz A.V., Kritchevsky S., Newman A.B. (2003). Association between regional adipose tissue distribution and both type 2 diabetes and impaired glucose tolerance in elderly men and women. Diabetes Care.

[6] Sironi A.M., Gastaldelli A., Mari A., Ciociaro D., Positano V., Buzzigoli E., Ghione S., Turchi S., Lombardi M., Ferrannini E. (2004). Visceral fat in hypertension: Influence on insulin resistance and beta-cell function. Hypertension.

[7] Goodpaster B.H., Krishnaswami S., Harris T.B., Katsiaras A., Kritchevsky S.B., Simonsick E.M., Nevitt M., Holvoet P., Newman A.B. (2005). Obesity, regional body fat distribution, and the metabolic syndrome in older men and women. Arch. Intern. Med..

[8] Lee S.W., Son J.Y., Kim J.M., Hwang S.S., Han J.S., Heo N.J. (2018). Body fat distribution is more predictive of all-cause mortality than overall adiposity. Diabetes Obes. Metab..

[9] Shuster A., Patlas M., Pinthus J.H., Mourtzakis M. (2012). The clinical importance of visceral adiposity: A critical review of methods for visceral adipose tissue analysis. Br. J. Radiol..

[10] Cheung A.S., de Rooy C., Hoermann R., Gianatti E.J., Hamilton E.J., Roff G., Zajac J.D., Grossmann M. (2016). Correlation of visceral adipose tissue measured by Lunar Prodigy dual X-ray absorptiometry with MRI and CT in older men. Int. J. Obes..

[11] Xia Y., Ergun D.L., Wacker W.K., Wang X., Davis C.E., Kaul S. (2014). Relationship between dual-energy X-ray absorptiometry volumetric assessment and X-ray computed tomography-derived single-slice measurement of visceral fat. J. Clin. Densitom..

[12] Kaul S., Rothney M.P., Peters D.M., Wacker W.K., Davis C.E., Shapiro M.D., Ergun D.L. (2012). Dual-energy X-ray absorptiometry for quantification of visceral fat. Obesity.

[13] Micklesfield L.K., Goedecke J.H., Punyanitya M., Wilson K.E., Kelly T.L. (2012). Dual-energy X-ray performs as well as clinical computed tomography for the measurement of visceral fat. Obesity.

[14] Choi Y.J., Seo Y.K., Lee E.J., Chung Y.S. (2015). Quantification of visceral fat using dual-energy X-ray absorptiometry and its reliability according to the amount of visceral fat in Korean adults. J. Clin. Densitom..

[15] Lin H., Yan H., Rao S., Xia M., Zhou Q., Xu H., Rothney M.P., Xia Y., Wacker W.K., Ergun D.L. (2013). Quantification of visceral adipose tissue using lunar dual-energy X-ray absorptiometry in Asian Chinese. Obesity.

[16] Svendsen O.L., Hassager C., Bergmann I., Christiansen C. (1993). Measurement of abdominal and intra-abdominal fat in postmenopausal women by dual energy X-ray absorptiometry and anthropometry: Comparison with computerized tomography. Int. J. Obes. Relat. Metab. Disord..

[17] Neeland I.J., Grundy S.M., Li X., Adams-Huet B., Vega G.L. (2016). Comparison of visceral fat mass measurement by dual-X-ray absorptiometry and magnetic resonance imaging in a multiethnic cohort: The Dallas Heart Study. Nutr. Diabetes.

[18] Kamel E.G., McNeill G., Han T.S., Smith F.W., Avenell A., Davidson L., Tothill P. (1999). Measurement of abdominal fat by magnetic resonance imaging, dual-energy X-ray absorptiometry and anthropometry in non-obese men and women. Int. J. Obes. Relat. Metab. Disord..

[19] Naboush A., Hamdy O. (2013). Measuring visceral and hepatic fat in clinical practice and clinical research. Endocr. Pract..

[20] Alberti K.G., Eckel R.H., Grundy S.M., Zimmet P.Z., Cleeman J.I., Donato K.A., Fruchart J.C., James W.P., Loria C.M., Smith S.C. (2009). Harmonizing the metabolic syndrome: A joint interim statement of the International Diabetes Federation Task Force on Epidemiology and Prevention; National Heart, Lung, and Blood Institute; American Heart Association; World Heart Federation; International Atherosclerosis Society; and International Association for the Study of Obesity. Circulation.

[21] Harrington D.M., Staiano A.E., Broyles S.T., Gupta A.K., Katzmarzyk P.T. (2013). Waist circumference measurement site does not affect relationships with visceral adiposity and cardiometabolic risk factors in children. Pediatr. Obes..

[22] Wang J., Thornton J.C., Bari S., Williamson B., Gallagher D., Heymsfield S.B., Horlick M., Kotler D., Laferrere B., Mayer L. (2003). Comparisons of waist circumferences measured at 4 sites. Am. J. Clin. Nutr..

[23] Bosy-Westphal A., Booke C.A., Blocker T., Kossel E., Goele K., Later W., Hitze B., Heller M., Gluer C.C., Muller M.J. (2010). Measurement site for waist circumference affects its accuracy as an index of visceral and abdominal subcutaneous fat in a Caucasian population. J. Nutr..

[24] World Health Organization (WHO) (2008). Waist Circumference and Waist-Hip Ratio: Report of a WHO Expert Consultation.

[25] Lohman T.G., Roche A.F., Martorell R. (1988). Anthropometric Standardization Reference Manual.

[26] Ross R., Berentzen T., Bradshaw A.J., Janssen I., Kahn H.S., Katzmarzyk P.T., Kuk J.L., Seidell J.C., Snijder M.B., Sorensen T.I. (2008). Does the relationship between waist circumference, morbidity and mortality depend on measurement protocol for waist circumference?. Obes. Rev..

[27] Rexrode K.M., Carey V.J., Hennekens C.H., Walters E.E., Colditz G.A., Stampfer M.J., Willett W.C., Manson J.E. (1998). Abdominal adiposity and coronary heart disease in women. JAMA.

[28] Zhu S., Wang Z., Heshka S., Heo M., Faith M.S., Heymsfield S.B. (2002). Waist circumference and obesity-associated risk factors among whites in the third National Health and Nutrition Examination Survey: Clinical action thresholds. Am. J. Clin. Nutr..

[29] Thomas G.N., Ho S.Y., Lam K.S., Janus E.D., Hedley A.J., Lam T.H. (2004). Impact of obesity and body fat distribution on cardiovascular risk factors in Hong Kong Chinese. Obes. Res..

[30] Jia W.P., Lu J.X., Xiang K.S., Bao Y.Q., Lu H.J., Chen L. (2003). Prediction of abdominal visceral obesity from body mass index, waist circumference and waist-hip ratio in Chinese adults: Receiver operating characteristic curves analysis. Biomed. Environ. Sci..

[31] Onat A., Avci G.S., Barlan M.M., Uyarel H., Uzunlar B., Sansoy V. (2004). Measures of abdominal obesity assessed for visceral adiposity and relation to coronary risk. Int. J. Obes. Relat. Metab. Disord..

[32] Hill J.O., Sidney S., Lewis C.E., Tolan K., Scherzinger A.L., Stamm E.R. (1999). Racial differences in amounts of visceral adipose tissue in young adults: The CARDIA (Coronary Artery Risk Development in Young Adults) study. Am. J. Clin. Nutr..

[33] Valsamakis G., Chetty R., Anwar A., Banerjee A.K., Barnett A., Kumar S. (2004). Association of simple anthropometric measures of obesity with visceral fat and the metabolic syndrome in male Caucasian and Indo-Asian subjects. Diabet. Med..

[34] Willis L.H., Slentz C.A., Houmard J.A., Johnson J.L., Duscha B.D., Aiken L.B., Kraus W.E. (2007). Minimal versus umbilical waist circumference measures as indicators of cardiovascular disease risk. Obesity.

[35] Rudolf M.C., Walker J., Cole T.J. (2007). What is the best way to measure waist circumference?. Int. J. Pediatr. Obes..

[36] Shen W., Chen J., Gantz M., Velasquez G., Punyanitya M., Heymsfield S.B. (2012). A single MRI slice does not accurately predict visceral and subcutaneous adipose tissue changes during weight loss. Obesity.

[37] Johnson S.T., Kuk J.L., Mackenzie K.A., Huang T.T., Rosychuk R.J., Ball G.D. (2010). Metabolic risk varies according to waist circumference measurement site in overweight boys and girls. J. Pediatr..

[38] Ambardar S., Cabot J., Cekic V., Baxter K., Arnell T.D., Forde K.A., Nihalani A., Whelan R.L. (2009). Abdominal wall dimensions and umbilical position vary widely with BMI and should be taken into account when choosing port locations. Surg. Endosc..

[39] Sabin M.A., Wong N., Campbell P., Lee K.J., McCallum Z., Werther G.A. (2014). Where should we measure waist circumference in clinically overweight and obese youth?. J. Paediatr. Child Health.

[40] Nazare J.A., Smith J., Borel A.L., Aschner P., Barter P., Van Gaal L., Tan C.E., Wittchen H.U., Matsuzawa Y., Kadowaki T. (2015). Usefulness of measuring both body mass index and waist circumference for the estimation of visceral adiposity and related cardiometabolic risk profile (from the INSPIRE ME IAA study). Am. J. Cardiol..

[41] Keys A., Fidanza F., Karvonen M.J., Kimura N., Taylor H.L. (2014). Indices of relative weight and obesity. Int. J. Epidemiol..

[42] Agarwal S.K., Misra A., Aggarwal P., Bardia A., Goel R., Vikram N.K., Wasir J.S., Hussain N., Ramachandran K., Pandey R.M. (2009). Waist circumference measurement by site, posture, respiratory phase, and meal time: Implications for methodology. Obesity.

